# Analysis of Hepatic Fibrosis Characteristics in Cirrhotic Patients with and without Hepatocellular Carcinoma by FTIR Spectral Imaging

**DOI:** 10.3390/molecules25184092

**Published:** 2020-09-07

**Authors:** Johanna Moreau, Pascaline Bouzy, Julien Guillard, Valérie Untereiner, Roselyne Garnotel, Aude Marchal, Cyril Gobinet, Christine Terryn, Ganesh D. Sockalingum, Gérard Thiéfin

**Affiliations:** 1 Université de Reims Champagne-Ardenne, BioSpecT-EA7506, UFR de Pharmacie, 51097 Reims, France; jhoannam@hotmail.fr (J.M.); pascaline.bouzy@gmail.com (P.B.); julien.guillard@univ-reims.fr (J.G.); roselyne.garnotel@univ-reims.fr (R.G.); amarchal@chu-reims.fr (A.M.); cyril.gobinet@univ-reims.fr (C.G.); ganesh.sockalingum@univ-reims.fr (G.D.S.); 2 Service d’Hépato-Gastroentérologie et de Cancérologie Digestive, Centre Hospitalier Universitaire de Reims, 51092 Reims, France; 3 Université de Reims Champagne-Ardenne, Plateforme en Imagerie Cellulaire et Tissulaire (PICT), 51097 Reims Cedex, France; valerie.untereiner@univ-reims.fr (V.U.); christine.terryn@univ-reims.fr (C.T.); 4 Laboratoire de Biochimie-Pharmacologie-Toxicologie, Centre Hospitalier Universitaire de Reims, 51092 Reims, France; 5 Service d’Anatomie et Cytologie Pathologique, Centre Hospitalier Universitaire de Reims, 51100 Reims, France

**Keywords:** FTIR imaging, k-means, fibrosis, collagen, cirrhosis, hepatocellular carcinoma

## Abstract

The evolution of cirrhosis is marked by quantitative and qualitative modifications of the fibrosis tissue and an increasing risk of complications such as hepatocellular carcinoma (HCC). Our purpose was to identify by FTIR imaging the spectral characteristics of hepatic fibrosis in cirrhotic patients with and without HCC. FTIR images were collected at projected pixel sizes of 25 and 2.7 μm from paraffinized hepatic tissues of five patients with uncomplicated cirrhosis and five cirrhotic patients with HCC and analyzed by k-means clustering. When compared to the adjacent histological section, the spectral clusters corresponding to hepatic fibrosis and regeneration nodules were easily identified. The fibrosis area estimated by FTIR imaging was correlated to that evaluated by digital image analysis of histological sections and was higher in patients with HCC compared to those without complications. Qualitative differences were also observed when fibrosis areas were specifically targeted at higher resolution. The partition in two clusters of the fibrosis tissue highlighted subtle differences in the spectral characteristics of the two groups of patients. These data show that the quantitative and qualitative changes of fibrosis tissue occurring during the course of cirrhosis are detectable by FTIR imaging, suggesting the possibility of subclassifying cirrhosis into different steps of severity.

## 1. Introduction

Cirrhosis is defined as an advanced stage of diffuse liver fibrosis in response to a chronic injury causing lobular disorganization with formation of fibrous septa and nodules of morphologically and functionally abnormal hepatocytes [1]. Classical histological scoring systems such as the Metavir [2] or Ishak systems [3] define cirrhosis as the ultimate stage of hepatic fibrosis but do not differentiate cirrhosis at different stages of severity. A subclassification of cirrhosis would be of clinical interest since cirrhosis at an early stage is potentially reversible when the causal factor is removed, whereas advanced cirrhosis may become irreversible [4] and progress to complications related to portal hypertension and hepatocellular insufficiency. There is also a risk of transformation into hepatocellular carcinoma (HCC). The identification of markers of advanced cirrhosis could allow a subclassification of cirrhotic patients, making it possible to select cirrhotic patients at higher risk and justifying a closer surveillance program.

A histological scoring system, the Laennec system [5,6], has been proposed to subclassify cirrhosis into three categories of severity according to the width of fibrous septa and size of nodules. Using this system, it has been shown that increasing severity of cirrhosis was tightly correlated with both the clinical stage of cirrhosis and the grade of portal hypertension [7]. In addition, an increasing incidence of liver related events has been reported according to the severity of cirrhosis as assessed by this scoring system [8,9]. In particular, the incidence of hepatic decompensation and HCC significantly correlated with the histological subclassification of cirrhosis [9]. However, this scoring system is semi-quantitative and not routinely used.

The molecular and structural characteristics of the extracellular matrix (ECM) vary during the dynamic process of hepatic fibrogenesis [4,10]. The production and the relative amounts of different types of collagen change during fibrosis development [4,11]. In parallel, the expression of tissue inhibitors of metalloproteinases (TIMPs) is upregulated and the expression of metalloproteinases (MMPs) decreased, thus favoring an imbalance between MMPs and TIMPs and facilitating the development of hepatic fibrosis [4,12]. Other ECM components may play a role in the progression of liver fibrosis. It has been shown that elastin monomers accumulate in liver tissue during fibrogenesis as a result of overproduction by myofibroblasts and a failure of its degradation by MMP12 [4]. In addition, post-translational modifications of collagen result in the formation of covalent intermolecular cross-links such as pyridinoline, which is involved in the stabilization of collagen at a late stage in the fibrotic process [13,14].

Spectral histopathology, using Fourier transform infrared (FTIR) imaging, provides a “vibrational fingerprint” of the tissue that reflects its molecular and structural composition [15]. Since the ECM molecular profile varies during hepatic fibrogenesis, we hypothesized that spectral analysis of fibrous septa in cirrhotic patients could allow a subclassification of cirrhosis, differentiating uncomplicated and complicated stages of the disease. It is well established that the different types of collagen have a specific spectral signature [16,17,18]. In addition, FTIR spectroscopic studies of collagen characteristics in rat tail tendons have revealed molecular changes related to the formation of collagen crosslinks during the aging of these tendons [19,20]. Previous studies have reported that FTIR imaging was able to differentiate regeneration nodules and fibrotic septa in human cirrhotic samples [21,22,23] and detect chemical modifications due to diabetes [24]. Thus, analysis of the FTIR spectral characteristics of hepatic fibrosis may have the potential to distinguish patients at different stages of cirrhosis.

The objective of this study was to analyze the spectral characteristics of hepatic fibrosis by FTIR imaging in cirrhotic patients, enabling the differentiation of patients with cirrhosis complicated by HCC and cirrhotic patients without complications.

## 2. Results and Discussion

### 2.1. Analysis of Spectral Images of Whole Hepatic Samples Acquired with a Projected Pixel Size of 25 µm

**Figure 1:**
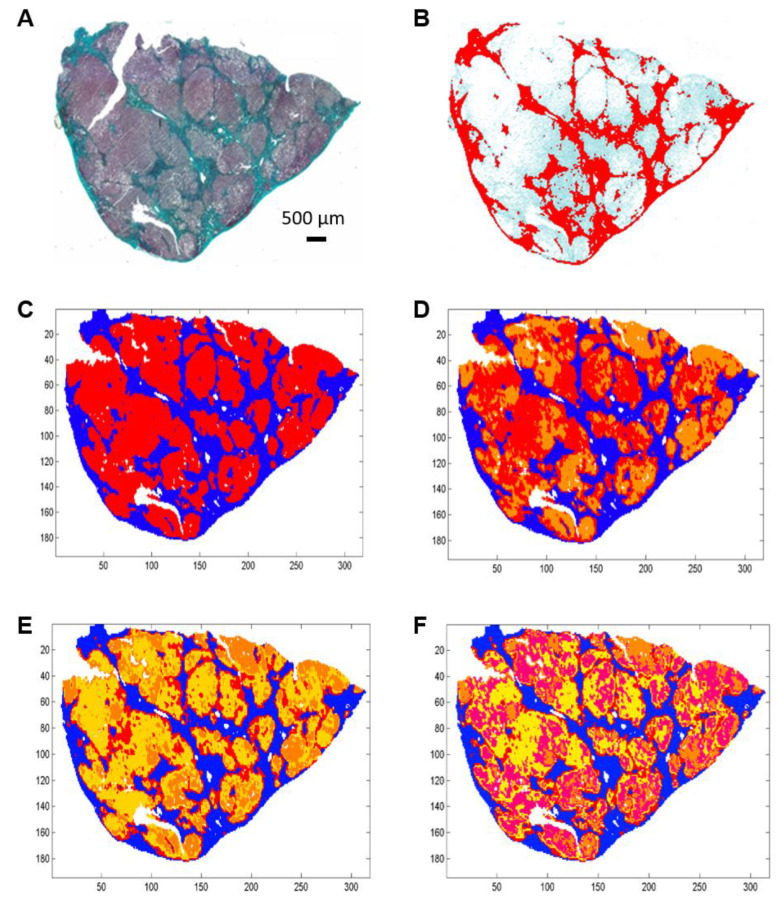
Example of histological and FTIR spectral images of an uncomplicated cirrhosis biopsy. (**A**) Histological section stained with Masson’s trichrome stain showing fibrosis in green. (**B**) Digital image analysis with fibrosis area in red. (**C**–**F**) K-means clustering of FTIR spectral images of an adjacent section in 2, 3, 4 and 5 clusters respectively from a 900–1800 cm^−1^ infrared absorbance dataset acquired with a projected pixel size of 25 µm. Clusters are displayed using random pseudo-colors. Fibrosis is represented by the dark blue cluster. Other colors correspond to regeneration nodules.

**Figure 2:**
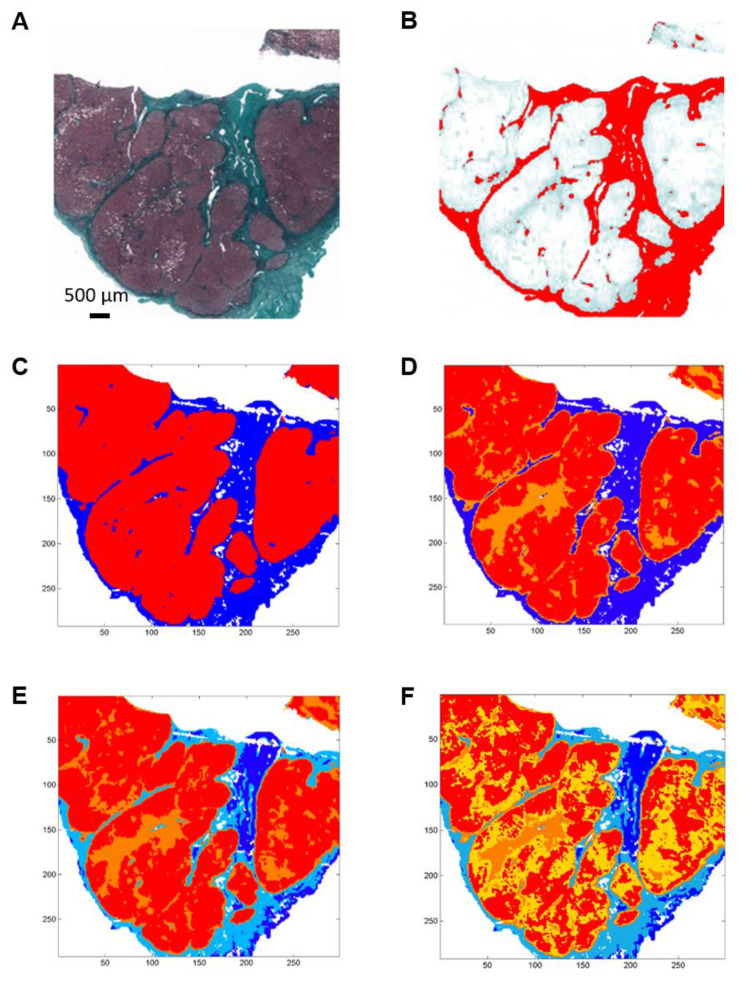
Example of histological and FTIR spectral images of a cirrhosis biopsy from a patient with hepatocellular carcinoma. (**A**) Histological section stained with Masson’s trichrome stain showing fibrosis in green. (**B**) Digital image analysis with fibrosis area in red. (**C**–**F**) K-means clustering of FTIR spectral images of an adjacent section in 2, 3, 4 and 5 clusters respectively from a 900–1800 cm^−1^ infrared absorbance dataset acquired with a projected pixel size of 25 µm. Clusters are displayed using random pseudo-colors. Fibrosis is represented by the dark blue cluster in C, D and in dark blue and light blue in E and F. Other colors correspond to regeneration nodules.

Histological and spectral images of two hepatic samples from patients with uncomplicated cirrhosis and cirrhosis complicated by HCC are presented as examples in Figure 1 and Figure 2.

Spectral images were partitioned into 2, 3, 4 and 5 clusters using the k-means method (Figure 1C–F and Figure 2C–F).

**Figure 3:**
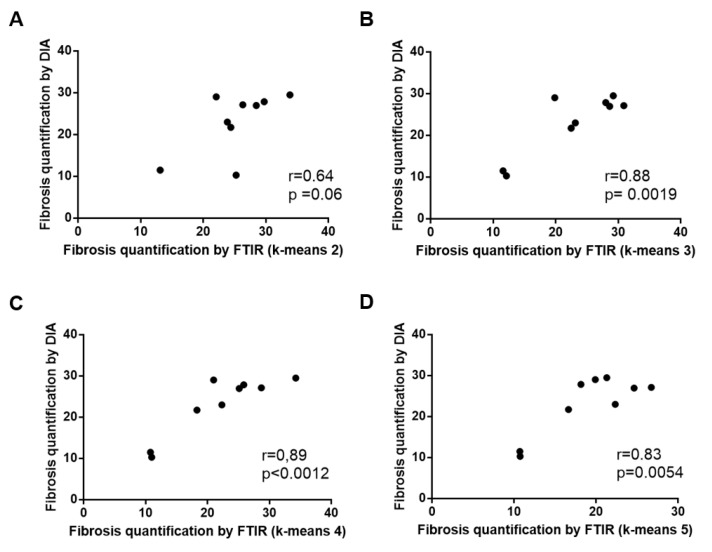
Correlations between fibrosis areas as measured by digital image analysis of histological sections after Masson’s trichrome staining and by k-means clustering of FTIR spectral images using 2, 3, 4 and 5 classes ((**A**–**D**) respectively). An outlier point corresponding to a fibrosis area of 60–70% was excluded from the analysis (see text and Supplementary Figure S1).

When compared to the adjacent histological section, the spectral classes corresponding to hepatic fibrosis and regeneration nodules were easily identified. Fibrosis tissue segregated out of nodules in the two-class partition and further partition delineated additional spectral subgroups, mainly in regeneration nodules. In contrast, fibrosis tissue remained homogeneous or mildly heterogeneous in up to two spectral classes compared to nodules as shown in Figure 1C–F and Figure 2C–F. The correlation analysis between fibrosis area percentages estimated by FTIR image clustering and by digital image analysis (DIA) was conducted after exclusion of an outlier (the point corresponding to a fibrosis area of 60–70%) in order to avoid a biased statistical analysis. The percentages of fibrosis area as estimated by FTIR image clustering with 3, 4 and 5 classes were significantly correlated to those evaluated by DIA of the corresponding histological sections stained with Masson’s trichrome as shown in Figure 1B and Figure 2B. The correlation coefficient r varied between 0.83 and 0.89 (*p*-value between 0.0012 and 0.0054) as shown in Figure 3. A similar tendency was observed when fibrosis areas were estimated by FTIR image clustering with two classes but the correlation did not reach the level of significance (*r* = 0.64, *p* = 0.06). For comparison, the scatter plots obtained using the whole dataset including the outlier are shown in the Supplementary Figure S1. The bias due to the outlier point explains the higher level of correlation. Altogether, our data suggest that FTIR image clustering with at least three classes may correctly differentiate between fibrosis tissue and regenerative nodules. However, this study was not designed to develop a predictive model. It is a proof-of-concept study and further work on a larger set of hepatic samples is necessary to build a classification model that would allow an automatic evaluation of fibrosis area without the need for stained adjacent histological sections.

This indicates that FTIR spectral analysis of liver tissue is an effective tool for quantifying the area of fibrosis with the advantages of being label-free and non-destructive. Previous studies have reported interest in FTIR spectral imaging to visualize fibrosis regions in hepatic tissues [21,22,23] but the correlation with quantification obtained by DIA of the stained sections has not been reported to date.

**Figure 4:**
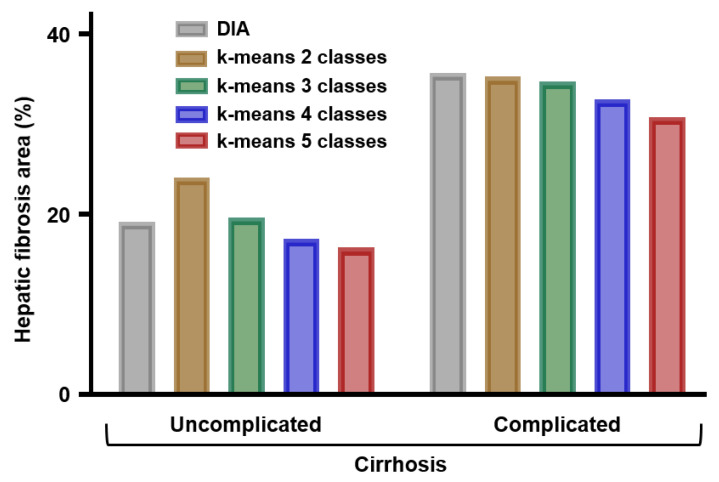
Percentages of fibrosis area in samples from uncomplicated (*n* = 5) and complicated cirrhosis (*n* = 5) as measured by digital image analysis (DIA) of histological sections or by k-means clustering of FTIR spectral images in 2, 3, 4, and 5 classes.

The mean percentage of fibrosis area in samples from patients with cirrhosis complicated by hepatocellular carcinoma was higher than in samples from patients with uncomplicated cirrhosis. This was observed with both methods of quantification, either DIA of stained histological sections or FTIR spectral imaging analysis based on the k-means method (Figure 4).

The small number of cases (*n* = 5 in each group) did not allow a statistical analysis but these data are in accordance with previous studies based on semi-quantitative and quantitative histopathological evaluations of hepatic fibrosis. The hepatic fibrosis area in cirrhotic tissues evaluated by the semi-quantitative histological method has been shown to be correlated with the clinical severity of the disease [7,8] and to be predictive of the risk of liver related complications including hepatocellular carcinoma [9] and of the risk of recurrence after resection of hepatocellular carcinoma [25,26]. In a comparative study, quantitative assessment of hepatic fibrosis by DIA was shown to have a better prognosis than semi-quantitative methods [27]. Thus, FTIR imaging of liver specimens appears as a reliable and easy-to-perform method for automatic quantification of liver fibrosis. Similar observations have been previously reported by our group for quantifying renal interstitial fibrosis in renal allograft biopsy specimens [28].

**Figure 5:**
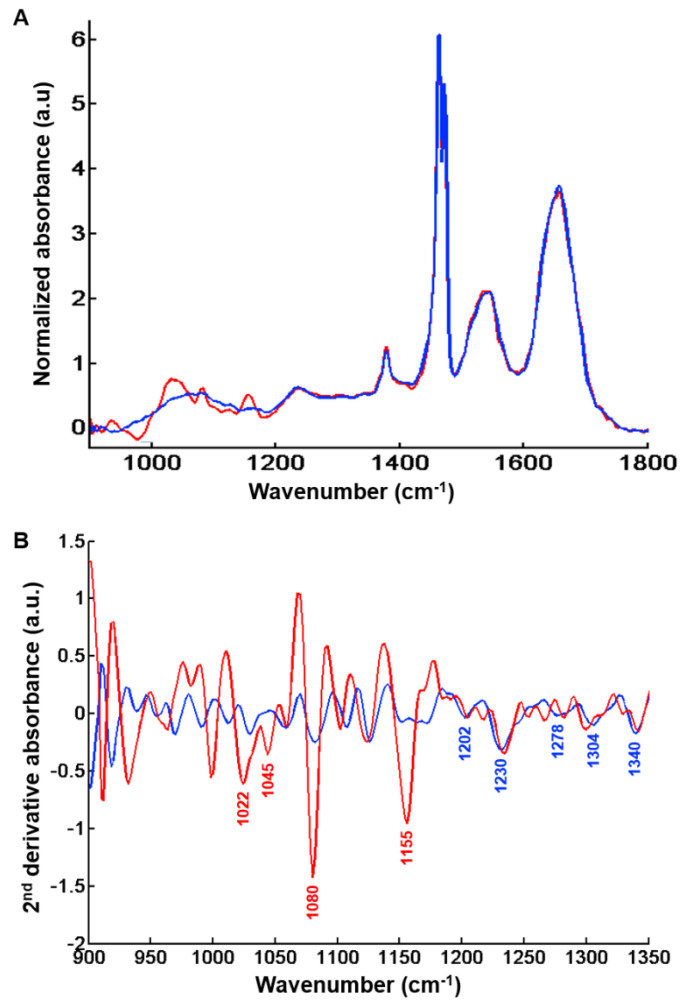
Centroids representative of regeneration nodules and hepatic fibrosis tissue after k-means clustering (*k* = 2) of the spectral image shown in Figure 1C. (**A**) Centroid spectra representative of fibrosis regions (dark blue line) and regeneration nodules (red line) in the 900–1800 cm^−1^ spectral range. (**B**) Second derivative of above centroid spectra and peak assignment with fibrosis (dark blue line) and regeneration nodules (red line) in the 900–1350 cm^−1^ spectral range. Peaks that can be tentatively assigned to glycogen (regeneration nodules) and collagen (fibrosis tissue) are indicated in red and blue respectively. Results are from FTIR images acquired with a projected pixel size of 25 μm.

Analysis of centroids from the k-means clustering with two classes highlights the spectral characteristics of regeneration nodules and fibrosis regions. By visual inspection, marked differences were observed between the two centroid spectra in the 900–1400 cm^−1^ range as illustrated in Figure 5A.

The centroids corresponding to regeneration nodules exhibit high absorbance levels related to the presence of glycogenated hepatocytes (Figure 5B). Differences in absorption features are mainly linked to glycogen, and observed at 1022 cm^−1^ (CH_2_OH group vibrations), 1045 cm^−1^ (C-O stretching coupled with C-O bending of the C-OH groups) [21,29,30,31], 1080 cm^−1^ (C-C stretch) [21,32,33] and 1155 cm^−1^ (C-O stretching vibration) [21,31,32,33].

The centroid spectrum corresponding to fibrosis tissue was characterized by absorbance peaks related to vibrational modes of collagen as evidenced by the peaks at 1174 cm^−1^ (C-O stretching) [34], 1202 cm^−1^, 1230 cm^−1^, 1278 cm^−1^, 1304 cm^−1^ (amide III) [32,35,36,37,38,39,40] and 1340 cm^−1^ (CH_2_ of collagen) [21,36,37,39,41].

### 2.2. Analysis of Spectral Images of Fibrosis Tissue Areas Acquired with a Projected Pixel Size of 2.7 µm

**Figure 6:**
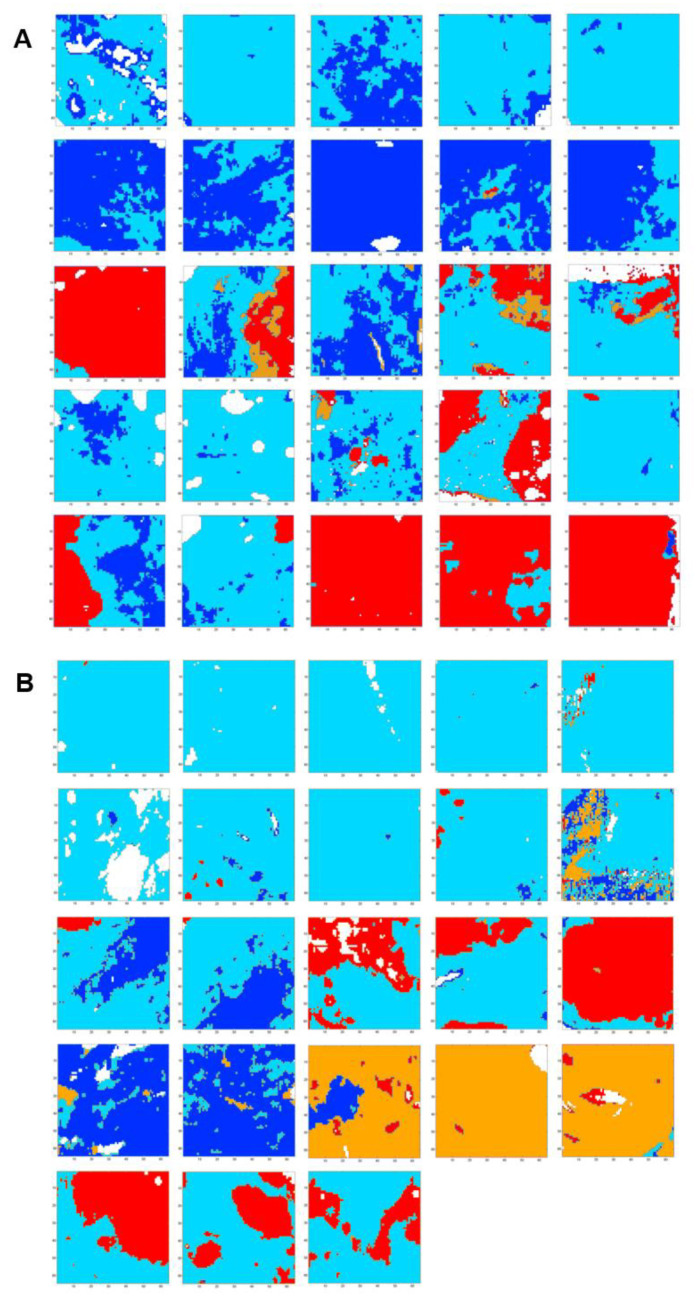
FTIR spectral images of 48 fibrosis areas (172.8 × 172.8 µm^2^) recorded from five samples of uncomplicated cirrhosis (panel (**A**): 25 images) and five samples of cirrhosis with HCC (panel (**B**): 23 images) clustered in four classes in the spectral range 1040–1425 cm^−1^. Results are from FTIR images acquired with a projected pixel size of 2.7 μm.

**Figure 7:**
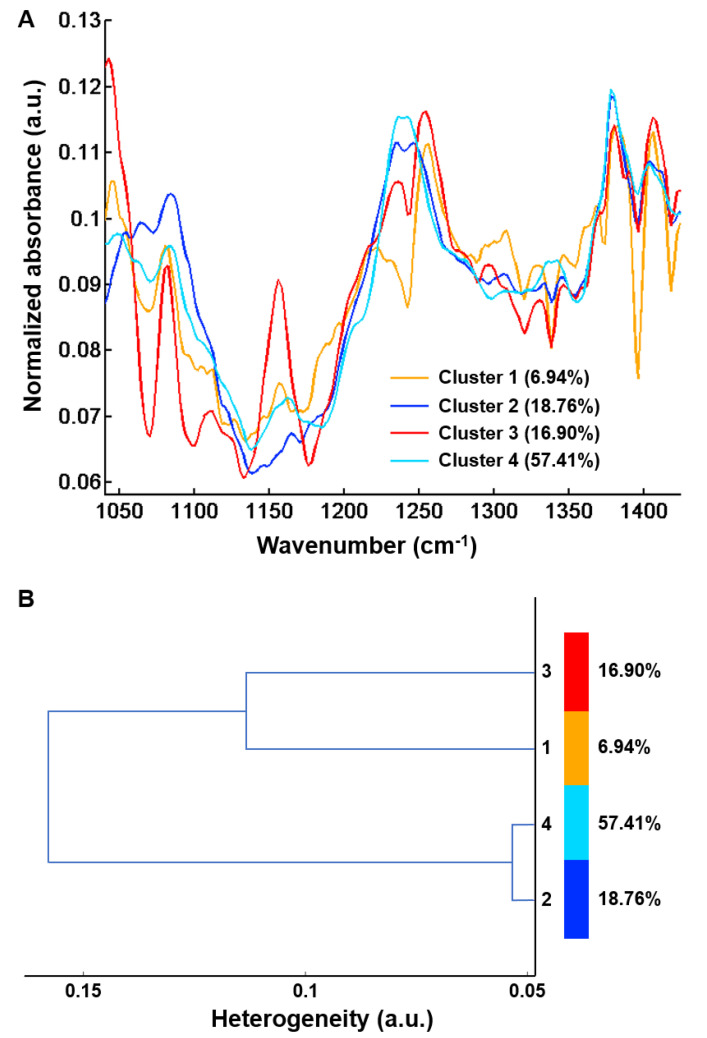
Centroids obtained after common k-means analysis of FTIR spectral images using four classes from 48 hepatic samples without (*n* = 25) and with (*n* = 23) complications in the 1040–1425 cm^−1^ spectral range. (**A**) Centroid spectra and (**B**) Hierarchical cluster analysis of the four centroid spectra. Results are from FTIR images acquired with a projected pixel size of 2.7 μm.

A common K-means clustering with four classes was carried out on 48 spectral images of fibrosis regions from the five samples of uncomplicated cirrhosis (25 spectral images) and five samples of cirrhosis with HCC (23 spectral images) on the 1040–1425 cm^−1^ spectral range. Spectral images are displayed in Figure 6 and cluster centroid spectra in Figure 7A.

**Figure 8:**
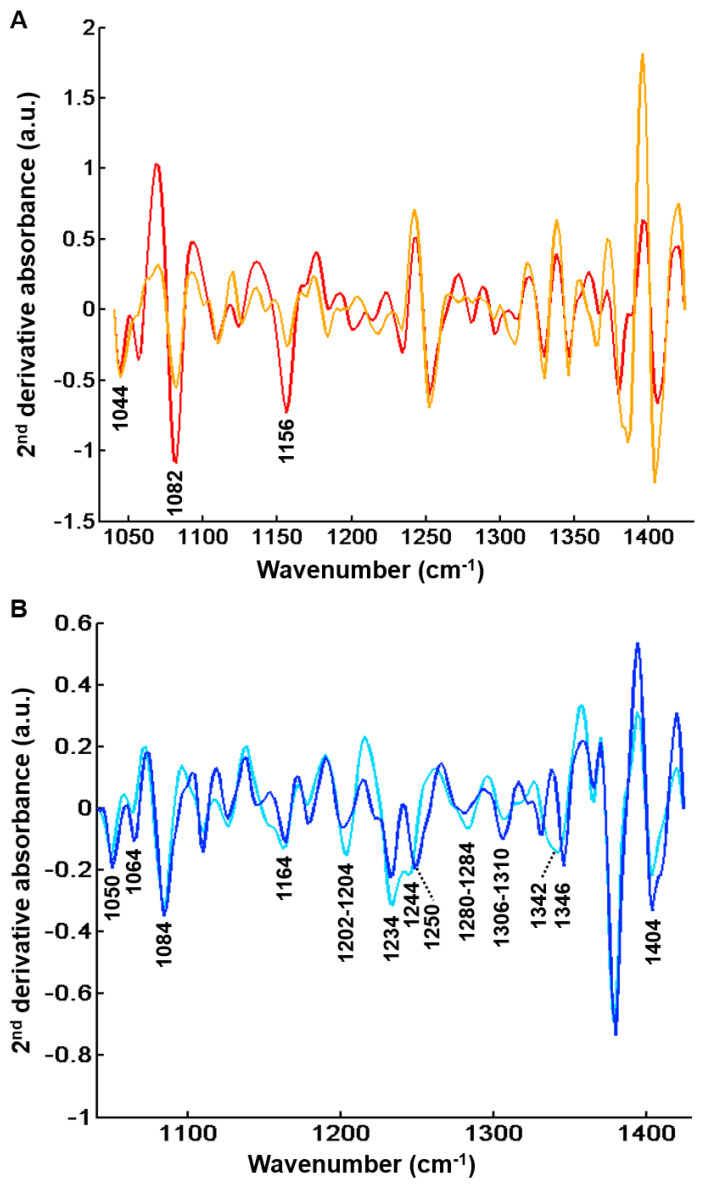
Second derivative centroid spectra after common k-means analysis (k = 4) of FTIR images from 48 hepatic samples without (*n* = 25) and with (*n* = 23) complications in the spectral range 1040–1425 cm^−1^. (**A**) Centroids corresponding to regeneration nodules. Peaks that can be tentatively assigned to glycogen are indicated (**B**) Centroids corresponding to fibrosis regions. Peaks that can be tentatively associated to collagen are indicated. Results are from FTIR images acquired with a projected pixel size of 2.7 μm.

Although images focused on fibrosis areas, some of them included parts of adjacent regenerative nodules. As shown in Figure 7B, the hierarchical clustering dendrogram clearly shows the formation of two groups. These groups were assigned by second derivative spectral analysis to regeneration nodules (clusters 1 and 3) and fibrosis (clusters 2 and 4) (Figure 8).

Clusters 1 and 3 displayed the characteristic absorption bands of glycogenated hepatocytes as described above at 1044 cm^−1^, 1082 cm^−1^ and 1156 cm^−1^ [29,30,31,32,33]. The two other centroid spectra (clusters 2 and 4) were related to the fibrotic tissue. These were characterized by high absorbance levels related to the presence of collagen at 1050 cm^−1^, 1064 cm^−1^, 1084 cm^−1^ (C-O and C-O-C stretching of carbohydrate moieties) [16], 1164 cm^−1^ (C-O stretching) [42], 1202–1204 cm^−1^, 1234 cm^−1^, 1280–1284 cm^−1^, 1306–1310 cm^−1^ (amide III) [32,35,36,37,38,39,40], 1342–1346 cm^−1^ (CH_2_ of collagen) [21,36,37,39,41] and 1404 cm^−1^ (CH_3_ of collagen) [16,38].

Of interest, the distribution of the two clusters within the fibrosis tissue appeared different between the two groups of liver specimens corresponding to early uncomplicated and late complicated stages of cirrhosis (Table 1). Although this is an interesting finding, the statistical analysis was considered inappropriate as the number of fibrosis areas was small and not equal for each patient, and it is therefore not presented.

Nevertheless, as shown in Table 1, the proportion of fibrosis tissue in cluster 4 was higher in complicated cirrhosis than in uncomplicated cirrhosis. Although definitive and statistically-based conclusions cannot be drawn, our results tend to support the hypothesis that fibrosis tissue in advanced complicated cirrhosis exhibits specific biomolecular and structural characteristics detectable by FTIR imaging that may be used to identify different stages of cirrhosis with increasing risk of clinical complications. These preliminary results are worth exploring in a larger data set. Compared with cluster 2, cluster 4 is characterized either by higher absorbance peaks or by a modification in spectral profile (1204, 1234, 1244, 1284 and 1342 cm^−1^). The higher absorption in amide III peaks (1204, 1234 and 1284 cm^−1^) may be explained by the remodeling of extracellular matrix with modifications of secondary structure of the collagen fibers. It is noteworthy that spectral modifications have been reported in an animal model of collagen aging using FTIR spectroscopy. It was shown that the spectral profile of collagen I from old rat tail tendons was characterized by an increase in absorption in the 1200–1300 cm^−1^ amide III region compared with newborns and young adults [19]. This is concordant with the hypothesis that changes in amide III peak absorption observed in cluster 4 may be related to a more advanced stage of fibrosis where crosslinking could modify the organization of collagen in extracellular matrix. In addition, spectral downshifts were observed at 1244 and 1342 cm^−1^ in cluster 4 compared with 1250 and 1346 cm^−1^ in cluster 2, respectively, which could also be related to structural and molecular constraints as a consequence of collagen crosslinks. Altogether, these results indicate that qualitative spectral changes reflecting the composition and structure of hepatic fibrosis could be useful markers of the severity of cirrhosis. With the advent of quantum cascade lasers for IR imaging and with an a priori knowledge of the specific spectral markers bands, these can be tuned to perform fast high-throughput screening of biopsies, thus overcoming the slow acquisition speed of FTIR-based infrared microscopes, which is a drawback for routine applications in clinics [21,43].

## 3. Materials and Methods

### 3.1. Selection and Preparation of Samples

The study was conducted on percutaneous liver biopsies and surgical liver samples from cirrhotic patients, stored in the tissue bank of the Laboratory of Biopathology of the Reims University Hospital. These samples were initially formalin fixed and paraffin embedded (FFPE) for routine histopathological analysis. Two groups of samples were selected: five from cirrhotic patients without complication with a follow-up of 10 years after the specimen collection and five from patients with cirrhosis complicated by an HCC. In patients with HCC, the study was performed on cirrhotic tissue located at a distance from the tumor.

Two adjacent 5 μm thick sections from selected FFPE blocks were obtained using a microtome (Leica Biosystem, Newcastle, UK). The first section was deposited on a glass slide for histology and the second on a calcium fluoride (CaF_2_) window for FTIR imaging. Patient records and tissue specimens were anonymized and de-identified prior to analysis. This procedure was conducted in accordance with the reference methodology MR004 of the “Commission Nationale de l’Informatique et des Libertés” (n°2206749, 13/09/2018).

### 3.2. Histological and Digital Analysis

For histology, Masson’s trichrome was used. It stains the cytoplasm in pink, the nuclei in black and highlights the collagen fibers in green (Figure 1A and Figure 2A). It was used to locate and quantify the area of fibrosis after digital scanning of these slides (iScan Coreo, Roche Ventana, Meillan, France) (Figure 1B and Figure 2B). The quantification was based on DIA using the public domain National Institutes of Health (NIH) Image program ImageJ (available at http://rsb.info.nih.gov/nih-image/). In this process, the histological image was binarized to eliminate the background of pure paraffin. Then, a color deconvolution in three channels was performed allowing the identification of collagen. The ratio of the fibrosis area to the total area gave the percentage of fibrosis in the sample.

### 3.3. FTIR Data Collection 

#### 3.3.1. Infrared Imaging of Whole Hepatic Samples

FTIR imaging acquisitions of whole hepatic samples were obtained using the Spectrum Spotlight^TM^ 400 microscope coupled to a Spectrum One FTIR spectrometer (Perkin Elmer, Villebon sur Yvette, France). This device was equipped with a liquid nitrogen-cooled, 16-pixel line mercury cadmium telluride (MCT) detector for imaging. The pixel size used was 25 × 25 μm^2^. Spectra were recorded in the 4000–650 cm^–1^ range with 8 accumulations per pixel at a resolution of 4 cm^−1^. Prior to image acquisition, a background spectrum was recorded on a clean surface of the CaF_2_ window with 90 accumulations at 4 cm^−1^ spectral resolution and automatically removed from each pixel spectrum using the Spectrum Image software (Perkin Elmer, Waltham, MA, USA). In addition, an image of pure paraffin was collected at the periphery of the tissue with the same parameters and used for digital deparaffinisation in the extended multiplicative signal correction (EMSC) model.

#### 3.3.2. FTIR Imaging Focused on Hepatic Fibrosis Tissue

In order to analyze more precisely the spectral characteristics of hepatic fibrosis, FTIR imaging was acquired with a projected pixel size of 2.7 μm in fibrosis tissue of each of the 10 samples. For this purpose, a Vertex 70 FTIR spectrometer coupled to a Hyperion 3000 microscope (Bruker Optics, Etlingen, Germany) was employed. It was equipped with a liquid nitrogen-cooled 64 × 64 pixels focal plane array MCT detector. Using a Cassegrain ×15 objective, a pixel resolution of 2.7 × 2.7 μm^2^ was obtained. Spectral images were acquired on the 3900–900 cm^−1^ range with 512 accumulations per spectrum at a resolution of 4 cm^−1^. As above, prior to image acquisition, a background spectrum was recorded on a clean surface of the CaF_2_ window with 512 accumulations at 4 cm^−1^ resolution and automatically removed from each pixel spectrum using the OPUS software (Version 7.2, Bruker Optics, Etlingen, Germany). For digital dewaxing, a pure paraffin image was collected at the periphery of the tissue using the same measurement conditions.

### 3.4. Spectral Data Preprocessing

Image preprocessing was performed using in-house algorithms written in Matlab (The Mathworks, Natick, MA, USA). Digital dewaxing was done by the EMSC method. This correction was applied to all spectral images in order to neutralize the paraffin spectrum in the IR spectra as previously reported by our group [44,45]. This EMSC model also eliminates outlier spectra, corrects scattering effects in FTIR spectra and normalizes them.

### 3.5. Analysis of Spectral Images of Whole Hepatic Samples

After the EMSC preprocessing, k-means clustering [46] was applied on FTIR images for regrouping the spectra according to their spectral similarity in k classes. In this procedure, each class is represented by a centroid spectrum that is the class mean spectrum. The k-means procedure is unsupervised and starts by selecting random k spectra as centroids and then allocating every spectrum of the spectral image to the nearest centroid using Euclidian distance as a metric. Each centroid is then readjusted and this operation is repeated until a stable partition is reached. To visualize the k-means clustering results, a different color is randomly assigned to each class and a reconstruction of the spectral image is performed. In our study, k-means clustering was used to partition each FTIR spectral image in 2, 3, 4 and 5 spectral classes using the 900–1800 cm^−1^ fingerprint region. All these spectral images were compared to their adjacent tissue section stained with Masson’s trichrome in order to annotate the spectral classes to their corresponding histological structures, particularly the fibrotic tissue. K-means clustering gave the percentage of the class(es) corresponding to fibrosis. Fibrosis area in histological sections was quantified by DIA as previously described.

In addition, the centroid spectra were analyzed to identify the discriminant IR absorption peaks corresponding to histological structures. They were derived to the second order to better visualize and assign overlapping peaks.

### 3.6. Analysis of Spectral Images Focused on Fibrosis Areas 

Comparison of spectral features of fibrosis between samples from uncomplicated and complicated cirrhosis was performed using spectral images with a higher spatial resolution. FTIR imaging with a pixel resolution of 2.7 × 2.7 μm^2^ was performed on fibrosis tissue of 172.8 × 172.8 µm^2^. Five fibrosis regions were selected for each sample except one where only three zones were available for analysis. Finally, 48 regions (25 from uncomplicated cirrhosis and 23 from complicated cirrhosis) were analyzed using a common k-means classification in four spectral classes on the 1040–1425 cm^−1^ spectral window, chosen as it includes most FTIR absorption peaks of collagen [20] and excludes paraffin peaks. In this procedure, spectral classes are common to all analyzed images, making it possible to compare the distribution of spectral characteristics between different samples. In common k-means image clustering, the same spectral class is represented by the same color coding.

### 3.7. Statistical Analysis

Pearson’s correlation coefficient was used to evaluate the correlation between the percentages of fibrosis areas in the whole tissue as measured on spectral images and on histological sections.

## 4. Conclusions

Our study shows that FTIR imaging of cirrhotic tissues allows the differentiation of regenerative nodules and fibrosis tissue and is effective for quantifying fibrosis area with the advantage of being label free and non-destructive. In addition, spectral image analysis with a higher spatial resolution highlights the qualitative changes of fibrosis tissue during the course of cirrhosis, differentiating liver fibrosis from complicated and uncomplicated cirrhosis. Future development would be to build a predictive model based on fibrosis tissue FTIR spectral characteristics allowing the subclassification of cirrhosis in different stages of severity corresponding to an increasing risk of complications such as HCC.

## Figures and Tables

**Table 1:** Comparison of the distribution of the two spectral classes corresponding to fibrosis areas from uncomplicated cirrhosis and cirrhosis with hepatocellular carcinoma (HCC).

	Fibrosis Areas (% of Total Tissue)	
**Spectral Classes of Fibrosis**	**Uncomplicated Cirrhosis**	**Cirrhosis with HCC**
**Cluster 4**	69.4%	83.9%
**Cluster 2**	30.6%	16.1%

## Supplementary Materials

The following are available online. Figure S1: Correlations between fibrosis areas as measured by digital image analysis of histological sections after Masson’s trichrome staining and by k-means clustering of FTIR spectral images using 2, 3, 4 and 5 classes (**A**, **B**, **C** and **D** respectively). The scatter plots include the outlier point corresponding to a fibrosis area of 60–70%.
