# Supplementary figures and images for: Analysis of Hepatic Fibrosis Characteristics in Cirrhotic Patients with and without Hepatocellular Carcinoma by FTIR Spectral Imaging
Source: Molecules. 2020 Sep 7;25(18):4092. doi: 10.3390/molecules25184092 (PMC7570752; doi:10.3390/molecules25184092)

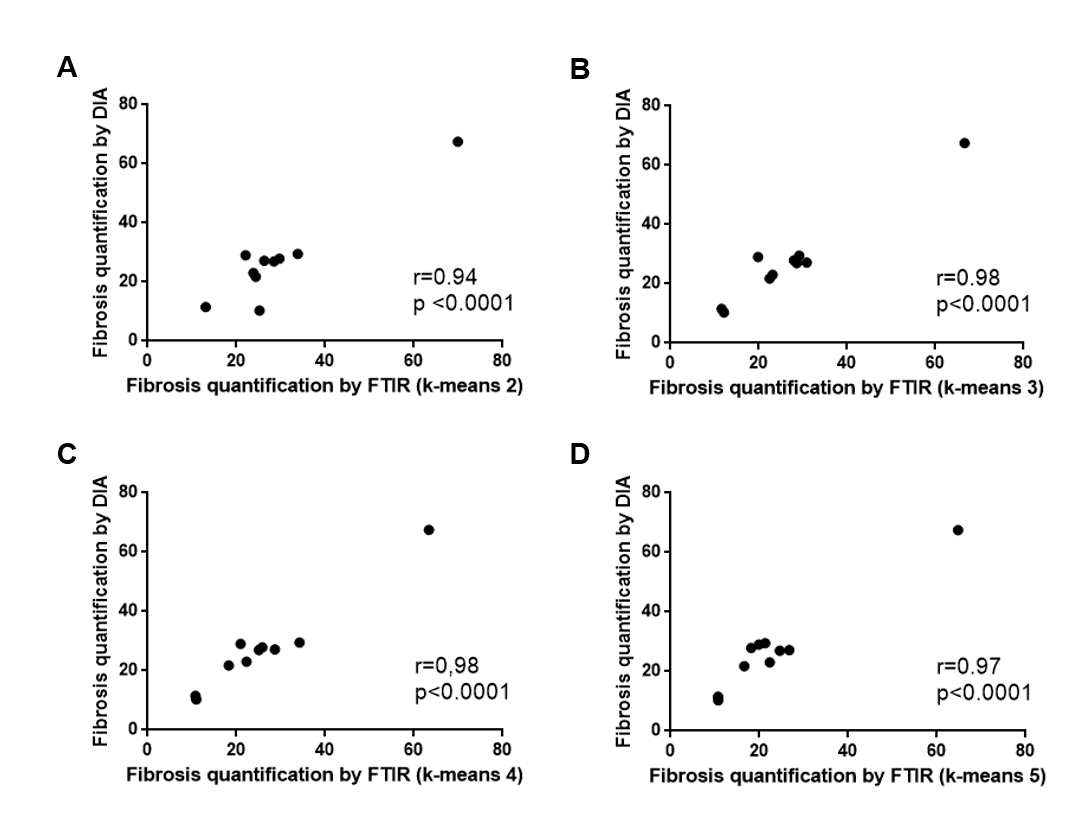

Supplement: Supplementary file 1 [file molecules-25-04092-s001.zip › molecules-889345-supplementary.tif]
